# Elucidating the interactions between the human gut microbiota and its host through metabolic modeling

**DOI:** 10.3389/fgene.2014.00086

**Published:** 2014-04-22

**Authors:** Saeed Shoaie, Jens Nielsen

**Affiliations:** Department of Chemical and Biological Engineering, Chalmers University of TechnologyGothenburg, Sweden

**Keywords:** gut microbiota, metabolic model, host metabolism, dietary modulation, SCFAs

## Abstract

Increased understanding of the interactions between the gut microbiota, diet and environmental effects may allow us to design efficient treatment strategies for addressing global health problems. Existence of symbiotic microorganisms in the human gut provides different functions for the host such as conversion of nutrients, training of the immune system, and resistance to pathogens. The gut microbiome also plays an influential role in maintaining human health, and it is a potential target for prevention and treatment of common disorders including obesity, type 2 diabetes, and atherosclerosis. Due to the extreme complexity of such disorders, it is necessary to develop mathematical models for deciphering the role of its individual elements as well as the entire system and such models may assist in better understanding of the interactions between the bacteria in the human gut and the host by use of genome-scale metabolic models (GEMs). Recently, GEMs have been employed to explore the interactions between predominant bacteria in the gut ecosystems. Additionally, these models enabled analysis of the contribution of each species to the overall metabolism of the microbiota through the integration of *omics* data. The outcome of these studies can be used for proposing optimal conditions for desired microbiome phenotypes. Here, we review the recent progress and challenges for elucidating the interactions between the human gut microbiota and host through metabolic modeling. We discuss how these models may provide scaffolds for analyzing high-throughput data, developing probiotics and prebiotics, evaluating the effects of probiotics and prebiotics and eventually designing clinical interventions.

## INTRODUCTION

The mammalian gut has been colonized with different types of microorganisms which has dynamic and beneficial symbiotic relationships. This metabolically active organ serves multiple functions such as assimilation of food that is indigestible by human cells and shaping of the immune system (Backhed et al., 2005). The microbes that inhabit our colon assist in ensuring resistance against different pathogens, while perturbations in metabolism of this complex ecosystem can cause different disorders (Kinross et al., 2011). To date, different studies have used DNA sequencing technology to depict the association of the gut microbiota with different complex diseases: type 2 diabetes (Qin et al., 2012; Karlsson et al., 2013b), obesity (Turnbaugh et al., 2006; Ridaura et al., 2013), and atherosclerosis (Karlsson et al., 2012). However, it has been reported that diet, age, environment, and ethnicity of the subjects have crucial impact on the microbial gut composition and these important factors should be accounted for during the conduction of association studies (Claesson et al., 2012). Historically, the study of microbial consortia has been restricted due to difficulties in culturing individual species. During the past few years, with the improvement of high-throughput technologies and culture-independent genomic methods, it has become possible to accurately characterize the composition of microbial ecology (Su et al., 2012). This has made it possible to understand the contribution of microbiota to different disorders through analyses of the species abundance (Handelsman, 2004).

**FIGURE 1:**
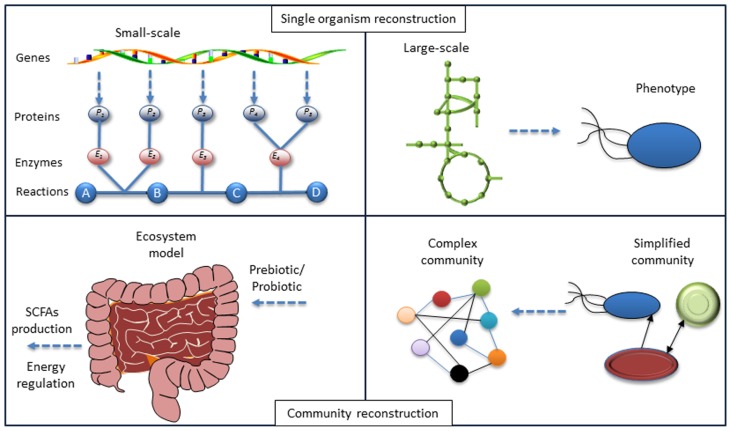
**Lessons from systems biology of single organisms to a community**. The model reconstruction for single organism based on genotype–phenotype connections has been developed. Identifying the local interactions between organisms in a community and the application of models for each organism-host interactome will enable the modeling of the microbiome and at the final step enable interpretation of the phenotype for the whole ecosystem. Hereby mathematical models can be used to test different hypotheses.

Metagenomics studies on the human gut have reported that the gut microbiota gene set is at least 150 times larger than the human gene set in a given individual (Qin et al., 2010; Arumugam et al., 2011). The numbers of species in the gut consortia can exceed 1000 while at least 160 species are common among individuals (Karlsson et al., 2013a). These studies suggest that the bacterial composition in the gut mainly belong to the phyla *Firmicutes* and *Bacteroides* (Qin et al., 2010; Huttenhower et al., 2012). The gut microbiota is also dominated by less abundant phyla such as *Proteobacteria*, *Actinobacteria*, and *Euryarchaeota* (Arumugam et al., 2011; Ridaura et al., 2013). Studying the interactions between these microbes in the consortia as well their interactions with the human host may enable us to elucidate the molecular mechanisms of interaction between the microbiome and the human host and eventually human diseases. This knowledge can also be applied to reveal the details of dysregulation in the gut microbiome (**Figure 1**). The major interactions between the gut microbes occur through the exchange of metabolites and the mediator of these interactions is the production of important metabolites, including short chain fatty acids (SCFAs; acetate, propionate, and butyrate; Ruppin et al., 1980). Species of *Roseburia*, *Eubacterium*, *Bacteroides,* and *Faecalibacterium* are examples of bacteria in the gut ecosystem that produces these metabolites (Holmes et al., 2012). SCFAs have potential effect on the host physiology since 60–90% of these SCFAs are absorbed by the epithelial cells (Ruppin et al., 1980). Thus, SCFAs regulate the energy supply for epithelial cells, control the pH in the colon and provide resistance to growth of pathogens (Gibson, 2004). Abnormalities in the metabolism of SCFAs lead to the occurrence of obesity, type2 diabetes, and colorectal cancer (Davie, 2003; Comalada et al., 2007; Sekhavat et al., 2007; Dumas, 2011).

Moreover, there are other metabolites that mediate the communication of the gut ecosystem with the human host. The interactions between microbe and host can be through the exchange of bile acids (Ridlon et al., 2006; Dawson et al., 2009), phenolic and aromatic acids (Lord and Bralley, 2008; Serino et al., 2012), cholines (Wang et al., 2011), fatty acids, and phospholipids (Serino et al., 2012). The primary bile acids, which are produced by the liver, are dehydroxylated by bacteria from the genus of *Lactobacillus*, *Bifidobacterium*, *Clostridium*, and *Bacteroides*. A small part of secondary bile acids is also absorbed by enterocytes which promote the lipid absorption and regulate the colonic energy homeostasis (Ridlon et al., 2006; Dawson et al., 2009). Choline is synthesized by *Faecalibacterium prausnitzii* and *Bifidobacterium* species and has a key role in lipid metabolism, and is implicated in liver and cardiovascular diseases (Wang et al., 2011). These microbial derived metabolites may also result in the dysregulation in the host by affecting the metabolism of different organs.

The synthesis of all these metabolites is strongly related to the composition of the microbiota as well as to the dietary pattern of each individual. The correlation of diet intake, composition of the gut microbiota and physiology of the host has been studied in animals and humans (Faith et al., 2011; Cotillard et al., 2013; David et al., 2014). Recently a study on dietary interventions and gene abundances in the gut microbiota of 38 obese and 11 overweight individuals was described. By taking up diet-induced weight-loss and weight stabilization interventions, a decrease in gene richness and differences in clinical phenotypes was observed (Cotillard et al., 2013).

To elucidate the interactions between the microbes in the gut ecosystems and further their interaction with the host, computational models can assist. In this context, genome-scale metabolic models (GEMs) can be employed to gain insights about the mechanistic details of the complex ecosystems and its interactions with the host (Mardinoglu et al., 2013a, 2014; Shoaie et al., 2013). GEMs provide a scaffold for integration and interpretation of high-throughput data to investigate the molecular details of such a community. Here, we review the latest progress on genome-scale metabolic modeling and how these models can be used to analyze the interactions between the gut ecosystem and the human host. We also discuss the elements of success toward whole body metabolism and increase our understanding of this complex system.

## GENOME-SCALE METABOLIC MODELS: A PLATFORM FOR INTEGRATION OF *OMICS* DATA

**FIGURE 2:**
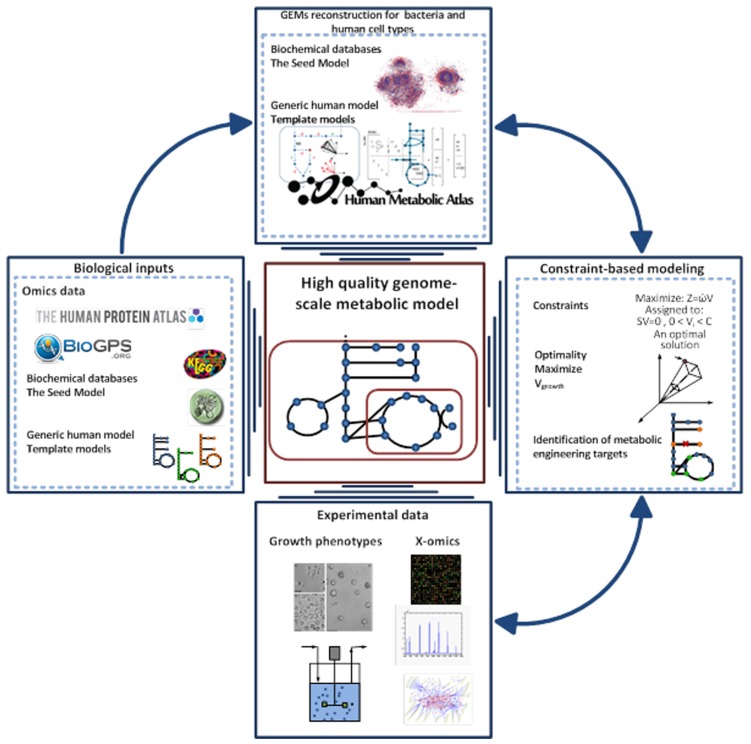
**Pipeline for high-quality GEM reconstruction**. This approach has been applied to the reconstruction of GEMs for microbes, tissue, and cell types. The Raven Toolbox is mainly applied for GEMs reconstruction based on template models and different databases ^35^. After having the draft model, sets of quality control can be applied to generate a fully functional model. This toolbox enables integration of *omics* data for more comprehensive analysis. It can generate the models in different formats, visualizing the simulations and overlaying the model in a metabolic map. Another powerful plug in is the INIT algorithm, which can be used for reconstruction of tissue-specific models based on a human generic metabolic model (Agren et al., 2012). The GEMs for tissues and microbes can be validated through constraint-based modeling approaches and available experimental data.

Over the last decade, the concept of predicting the phenotype of single organisms from their genotype using GEMs has been well established (Fleischmann et al., 1995; Edwards and Palsson, 1999). This type of computational models has been used to describe the molecular mechanism of the organism under study based on genome annotation, biochemical reaction databases, and literature reviewing. GEMs are the collection of bio-chemical reactions and associated genes, which indicate the existence of proteins in the target organism (Price et al., 2004; Liu et al., 2010). The manual construction of GEMs is time consuming and laborious, so different approaches have been generated to automate the reconstruction process (Henry et al., 2010; Liao et al., 2012; Agren et al., 2013). Among them, the RAVEN toolbox was recently described, which has the capability to reconstruct a model based on homology or sequence of the target organism (Agren et al., 2013). Gap-filling and quality control procedures are also included in the toolbox that thereby enables generation of connected models in an automated fashion. The RAVEN toolbox has been used for reconstructions of several GEMs such as *Pichia stipitis* (Caspeta et al., 2012), *Saccharomyces cerevisiae* (Osterlund et al., 2013), and *Penicillium chrysogenum* (Agren et al., 2013). The quality control step of the toolbox allows for consistency check of the models with experimental data. This step is done with the impact of imposing different constraints such as thermodynamics and secretion and uptake fluxes can be evaluated. Finally the RAVEN toolbox allows for contextualizing *omics*-data, setting up hypothesis in metabolic engineering and studying the interactions of organisms by network-based discovery (Oberhardt et al., 2009; **Figure 2**).

The computational methods for studying the metabolism of single organisms has been developed and applied successfully (Thiele and Palsson, 2010). GEMs were traditionally limited to metabolism, but there are large portions of the genome that encode for proteins involved in translation, transcription, signaling, and replication (Gil et al., 2004). Recently macromolecular expression data have been integrated with GEMs to present improved description of the target organism (Lerman et al., 2012). The progress in sequencing technology has allowed us to unravel the molecular mechanisms of complex communities (Zomorrodi and Maranas, 2012). The GEMs for each organism may assist to gain insight about the different metabolic interactions, identify the specific metabolism of each species and set hypothesis for finding the optimal conditions for the community (Stolyar et al., 2007; Shoaie et al., 2013). In addition, community modeling can be used to find the correlation between the individuals of each community and detailed mechanisms behind these communities, enabling studies of the gut microbiome.

## METABOLIC MODELING OF GUT MICROBIOTA

Setting up a metabolic model for each species and integrating these models may allow us to study the overall function of a microbial community. Metagenomics studies can quantify the relative abundance of each species in a community but it does not enable description of the function of each individual. Abubucker et al. (2012) have used the outcome of metagenomics studies to reconstruct metabolic models for each abundant species. The content of these metabolic models were similar to each other, but they had diverse functions for different environmental conditions. While it comes to metabolic modeling of communities, the interactions between the species has a key role in shaping of the consortia. Predictive methods have been established to delineate the interactions between bacterial species. In this context, 118 bacterial metabolic models have been deposited (Freilich et al., 2011) into the Seed database (Henry et al., 2010). Through the possibility of a co-growth concept for the community, three types of conditions were introduced: (i) no interaction (the species don’t have overlapping substrates), (ii) competition (species are competing for the same substrates resulting in higher growth for one species), and (iii) cooperation (the overall growth increase with positive interactions between species; Freilich et al., 2011). The distribution values for competition and cooperation across the species were identified by implementing constraint-based modeling, setting growth as an objective function and predicting a competition-inducing media. In a recent study, 154 metabolic models of species have been used to understand the competition and complementarity of species by means of co-occurrence of species (Levy and Borenstein, 2013). It has been reported that co-occurrence of competitive species is more frequent among the individuals. Although this generic method assists in delineating the interactome between a large number of metabolic models of bacteria, the correct set of metabolites in the media and the lack of functionality in these metabolic models decrease the reliability of the method for understanding the interaction between species and further the identification of the role of single species in the overall metabolism.

In order to have an increased understanding of the metabolism in microbial communities, Shoaie et al. (2013) studied the gut ecosystem based on previously published high-throughput metagenomics studies (Samuel and Gordon, 2006; Mahowald et al., 2009). Phylogenetic information was collected and three species were chosen as the representative of the three different abundant phyla in the gut microbiota (Qin et al., 2010; Arumugam et al., 2011). Functional GEMs for three bacteria were reconstructed based on different biochemical reaction databases and intensive manual curation. *Bacteroides thetaiotamicron* from the *Bacteroides* phyla and *Eubacterium rectale* from the Firmicutes phyla were selected since these two phyla are the most abundant in the human gut. *Methanobrevibacter smithii* as a dominant archaeon, which has a capability to mediate hydrogen metabolism, was also chosen to simulate the interactions between these three species. Three GEMs including *iBth1201*, *iEre400* and *iMsi385* were validated individually and used for prediction of each specie’s contribution to the metabolism of the mono-colonized germ-free mice (Samuel and Gordon, 2006; Mahowald et al., 2009). First, the uptake of substrates and secreted by-products were predicted and compared with experimental values by constraining the biomass and using a minimization algorithm. In metabolic modeling of the ecosystem with two species, the main exchangeable metabolite between the *B. thetaiotamicron* and *E. rectale* was found to be acetate. It was observed that the butyrate level in the ecosystem increased due to the mediation of acetate between the species. After partly absorption of these two SCFAs by the epithelial cells, their predicted levels were in agreement with the measured values in co-colonized germ-free mice experiments. It was also shown that glucan was mainly consumed by *B. thetaiotamicron* as a main substrate. Next, the interactions of three species were studied in the gut ecosystem through metabolic modeling. The simulations were performed based on maximization of the community biomass while the substrate was fixed and the SCFAs profile was predicted. It was observed that there was competition for acetate uptake between *E. rectale* and *M. smithii,* while the major part of CO_2_ and H_2_ were consumed by *M. smithii* and converted to CH_4_ through methanogenesis.

**FIGURE 3:**
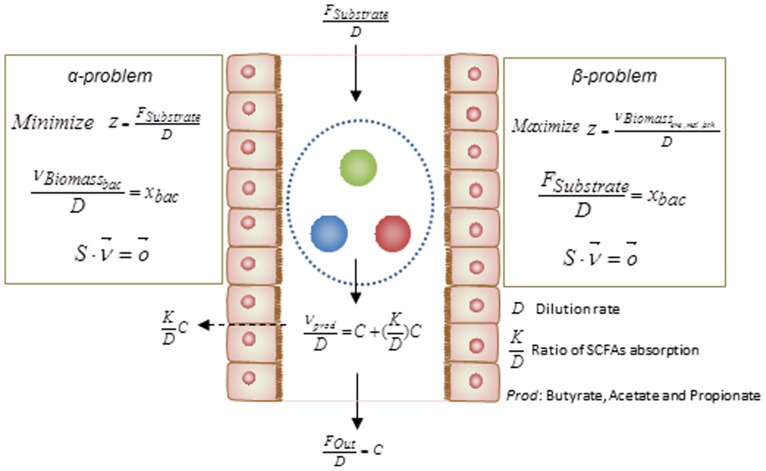
**Mathematical formulations for metabolic modeling of the gut microbiota**. The formulations of the α- and β-problem have been defined. In the α-problem the abundance of each individual species in the community is well-defined and through minimization for substrate/diet, the profile of SCFAs is estimated. In the β-problem the abundances of individuals can be predicted through maximization of the community biomass and by using a well-defined substrate/diet composition. (*D: dilution rate*, $\frac{K}{D}$: *ratio of* SCFAs *absorption, Prod: butyrate, propionate, and acetate*).

Basically, the above simulations have been categorized as two different mathematical formulation referred as α and β problems. These two types of simulation can assist to test hypotheses regarding the occurrence of metabolic abnormalities. By knowing the relative abundance of gut bacteria for specific disorders, solving the α problem enables prediction of the profile of secreted SCFAs, i.e., this method simulates the secretion of metabolites as a function of bacterial abundance. However in order to test the impact of diet composition on the gut microbiota and its association to metabolic disorders, solving the β problem may assist in estimation of the relative abundances of each bacteria in the gut ecosystem. Integrating the result of the analysis with different metabolic disorders associated with the gut microbiome would facilitate the design of diet as a key factor in shaping the gut bacterial composition. The formulations of these two types of problem are depicted in details in **Figure 3**.

The existence of the transcriptomics data for two cases for the presence/absence of *B. thetaiotamicron* and *E. rectale* in mono-colonized germ-free mice ^52^ was used to reveal transcriptional regulation at the gene and metabolite levels. Adaption of *E. rectale* to *B. thetaiotamicron* was found to be mediated through up-regulation of the genes associated with the TCA cycle, purine and pyrimidine metabolism, and down-regulation of genes associated with the carbohydrate metabolism in *E. rectale.* This adaptation showed that, during growth, of *E. rectale* shifted to utilization of amino acids, in particular glutamine, in the presence of *B. thetaiotamicron*. It was proposed that this shift may be the reason for a drop in the plasma glutamine level of obese mice that have an increased abundance of Firmicutes. This observation has been confirmed by a recent study where a colonization of germ-free mice with a culture collection from obese mice resulted in an increase in the metabolism of leucine, isoleucine, and valine (Ridaura et al., 2013). In order to understand the effect of the gut microbiata to the host metabolism in health and disease states, a whole body model should be reconstructed. In this context, models for each cell/tissue type and each species in the gut should be integrated and used for the simulation of the metabolism using a holistic approach.

## MODULATION OF GUT MICROBIOTA-HOST METABOLIC INTERACTIONS

There are clear functional links between the gut microbiota and its host that may lead to increase in the harvested energy and alterations in the host metabolism. Some of the interactions of host and gut microbiota were summarized in the introduction section. Here, we will discuss the recent progresses in constraint-based modeling of different cell/tissue types in the human body and the steps toward the integration of these models with models for the gut ecosystem. The efforts for modeling of the human metabolism started with the reconstruction of generic human models including Edinburgh human metabolic model (EHMN; Ma et al., 2007) and *Recon1* (Duarte et al., 2007). These literature based models represent comprehensive collections of biochemical reactions that occur in the human body. Recently, generic models like the human metabolic reaction (HMR; Agren et al., 2012) and *Recon2* (Thiele et al., 2013), that both integrate components of EHMN and *Recon1*, have been published. These generic models are useful resources for computational modeling as well as network dependent analysis. However, metabolism varies in each tissue of the human body and it is therefore necessary to reconstruct cell or tissue specific models. In this context, the INIT (integrative network inference for tissues) algorithm (Agren et al., 2012, 2014) and several other algorithms [reviewed in Mardinoglu et al. (2013b)] have been developed to generate cell/tissue specific draft GEMs based on generic human models and high-throughput data, e.g., proteomics data from the Human Protein Atlas (Uhlen et al., 2010; Fagerberg et al., 2014; **Figure 2**). Recently, several functional cell type specific GEMs have been generated for liver (Mardinoglu et al., 2014), brain (Lewis et al., 2010), adipocyte (Mardinoglu et al., 2013a), and alveolar macrophage (Bordbar et al., 2010) using semi-automated approaches.

It is feasible to understand more about whole body physiology by studying the interactions between the functional cell/tissue specific models and integration of clinical data (Mardinoglu and Nielsen, 2012). This knowledge can be used to elucidate the progression of metabolic disease, make hypothesis for new therapies and design new clinical interventions. To this end, the intercellular interactions between three metabolically active human cell types including adipocytes, hepatocytes, and myocytes have been studied and these models were used for integration of high-throughput data to find metabolic variations and reaction activities (Bordbar et al., 2011). Furthermore, the modeling of the interactions between different organs has provided an increased understanding about the stages of metabolic disorders.

**FIGURE 4:**
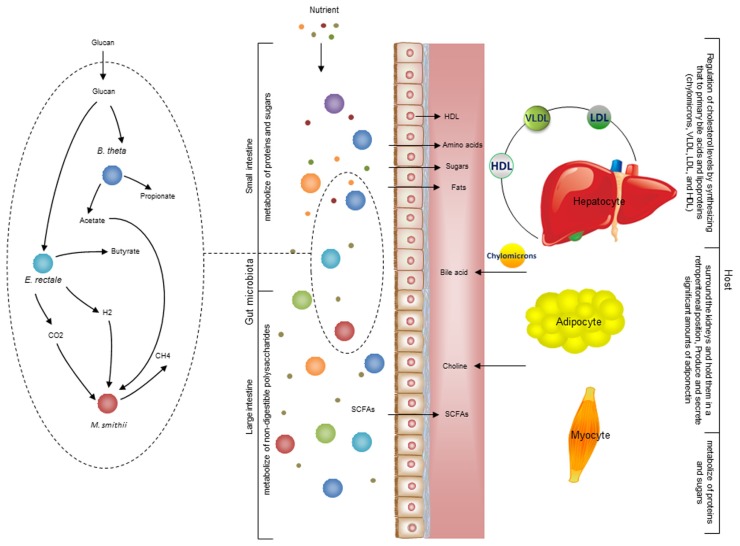
**Interaction between the gut microbiota and host**. There are different types of metabolic interactions between the bacteria in the gut ecosystem. A simplified model community including three species where *B. thetaomicron* and *E. rectale* consume oligo and poly-saccharides, and *M. smithii* takes up CO_2_ or formate, and acetate. The primary interactions in this simplified community involve acetate, H_2_, and CO_2_. The primary products are three SCFAs: acetate, propionate, and butyrate. These metabolites are mainly absorbed by epithelial cells. Butyrate absorbed by colonocytes for energy, while propionate and acetate are transferred to the portal vein and from there to other cell types, including adipocyte and hepatocyte. The micronutrients are digested in stages as food travels through sections of the gut. Some carbohydrates, proteins, and fats are digested by host enzymes and indigestible ones are degraded by the microbiota. This process initiates mainly in the stomach and continues significantly through the small and large intestine. The available SCFAs are transported to liver through the portal vein. Since hepatocyte regulate cholesterol levels by synthesizing primary bile acids and lipoproteins [chylomicrons, very low-density lipoprotein (VLDL), low-density lipoprotein (LDL), and high-density lipoprotein (HDL)] ^28^. It is very likely that the production of acetate and other compounds by the microbiome profoundly impacts this regulation. There is also a crosstalk between adipocytes and myocytes through free fatty acid transport. Understanding these interactions between organs is necessary to overcome the complexity of metabolic modeling the interaction between host and gut microbiota.

There is also metabolic host- bacterial symbiosis for instance in the small intestine and colon (**Figure 4**). The butyrate produced by bacterial fermentation in the lumen, provides energy for colonic epithelium (Waterman, 1996). In addition to epithelial and enterocyte cell types in the small and large intestine, the intestinal macrophages also play a key role for maintaining homeostasis between the host and the bacterial community. Macrophages are present in large populations in the human intestine and can mainly be found in the mucosa. Macrophages have been implicated as the cause of inflammatory bowel diseases and as a target for treatment (Mowat and Bain, 2011). Previously, a cell specific alveolar macrophage metabolic model has been reconstructed and its interaction with pathogenic bacteria has been studied (Bordbar et al., 2010). This model can be used as a draft model to reconstruct a model for macrophages that exist in the intestine and it can eventually be used to elucidate the interactions between bacterial ecosystems, the mucus layer and intestinal epithelial cells.

A metabolic model for human small intestinal enterocytes has been reconstructed (Sahoo and Thiele, 2013). Pathways for metabolism of carbohydrates, amino acid, dietary fibers, and lipids were the most occurring in this model. The effect of an American and balanced diet was studied with this model and different flux distributions were predicted based on the diet. An American diet is mainly enriched in calories and the certain essential nutrients are low compared with a balanced diet (Flock and Kris-Etherton, 2011). By comparing an American to a balanced diet, higher flux values for conversion of glutamine to glucose, proline, ornithine, and citrulline were found for a typical American diet whereas lower flux values for synthesis of 5-methyl-tetrahydrofolate were predicted through the use of this model (Sahoo and Thiele, 2013).

## EFFECT OF DIET ON COMPOSITION OF GUT MICROBIOTA

The composition of bacteria in the gut ecosystem is significantly influenced by the diet (David et al., 2014). The three primary macronutrients that affect the microbiota and host are carbohydrates, proteins, and fats (Scott et al., 2013). Carbohydrates that cannot be digested by the host are fermented by the gut microbiota, e.g., resistant starch that is mainly utilized by *Ruminococcus bromii* (Daniel et al., 2014). Around 40 gram of carbohydrates, which consist of resistant starch, non-starch polysaccharides and oligosaccharides, reach the colon on a daily basis and is here partially fermented by the gut microbiota. The fermentation of protein is also located in the distal compartment of the colon and 12–18 g proteins reach the colon each day (Scott et al., 2013). The *Bacteroides* and *Clostridium* species are examples of predominant bacteria for protein fermentation (Macfarlane et al., 1986) and they mainly use protein for production of the three major SCFAs, ammonia, phenols, amines, and sulfides (Hamer et al., 2012). The third macronutrient that is mainly available in the diet is fat, which is mostly absorbed through the small intestine and only a small percentage is excreted in the feces (Gabert et al., 2011). As it has been studied before, the production of SCFAs are decreased with a low fat diet compared to a high fat diet (Brinkworth et al., 2009), while from different mouse studies it has been concluded that, the shift in the composition of microbiota for different fat diets is not related to the host phenotype (Zhang et al., 2012).

Metagenomics studies based on ethnicity have shown some evidence about the association of the long-term diet and the composition of bacteria in the gut. The studies of fecal samples of European adults are clustered together with American adults, while Malawians and African are separated from them and clustered together (Yatsunenko et al., 2012). Their diet information also indicated the separation between the groups since African and Malawians diet is rich on plant polysaccharide whereas American and European diet is rich on proteins. Another study on fecal samples of rural African and urban Italian children showed significant differences in the abundance of *Bacteroidetes* that consume proteins (De Filippo et al., 2010). This diversity was ascribed to the differences in the micronutrients contents of the African and Italian diet.

Based on different diet composition, it is possible to design non-viable food components that modulate the composition of gut microbiota resulting in benefits for the host metabolism (Scott et al., 2013). Previous studies have shown the effect of different prebiotics on the human gut microbiota (Kleessen et al., 1997). The prebiotic chicory inulin resulted in a decrease of *Enterobacteria* and increase of *Bifidobacteria* species in the gut microbiota of elderly human (Kleessen et al., 1997; Biagi et al., 2012). In another study, using the galacto-oligosaccharides as a prebiotic caused an increase in *Bifidobacteria* species and increased the level of butyrate (Walton et al., 2012). The design of the prebiotics can be improved through the use of metabolic modeling that accounts for the interactions between the host and gut microbiota. Gathering the information about substrates utilized by bacteria and host tissues may enable the testing of different dietary hypotheses and the rational design of prebiotics.

## CONCLUDING REMARKS

As discussed above, it is feasible to understand the whole body metabolism by studying the interactions between different cell types/tissues and microbial GEMs. Genome-scale modeling may facilitate the comprehensive analysis of clinical data and assist in unraveling the mechanisms behind different complex disorders. But to reach this goal, it is necessary to develop new mathematical formulations, algorithms, and integrate these tools with constraint-based modeling. Systems-level or global objective functions should also be formulated for predicting the phenotype of the gut ecosystem. This has been modeled for the simplified community that is comprised of three microorganisms (Zomorrodi and Maranas, 2012). The dissipation of energy can be applied as a global objective function to model the whole body metabolism since the growth cannot be used as an objective function for most human cell types. The minimization of the energy dissipation for host, maximizing growth for microbe can be considered as an individual objective functions in metabolic modeling. By defining a global objective function based on localized metabolic interactomes of these individuals, it may be make it possible to quantitatively describe the interactions and eventually predict the overall flux distributions in the human body. This approach may assist in generating new hypotheses about the contribution of single or a community of microbes to the overall human metabolism. Applying the high-throughput technologies on the collected blood, stool, urine samples and detecting significant changes in the metabolite levels may facilitate improvement of the functional accuracy of the reconstructed models. This concept can also be expanded for the generation of the personalized GEMs for tissue/cell types and microbiota, which may contribute to the development of personalized medicine (Hood et al., 2012; Nielsen, 2012; Agren et al., 2014). Conceptualization of personalized GEMs may enable us to reduce the time and cost of clinical studies and assist to predict targets and potential treatment for each specific patient.

## Conflict of Interest Statement

Jens Nielsen is a shareholder of MetaboGen AB. Saeed Shoaie declares no competing financial interests.
